# Emotional Intelligence and Creative Self-Efficacy among Gifted Children: Mediating Effect of Self-Esteem and Moderating Effect of Gender

**DOI:** 10.3390/jintelligence11010017

**Published:** 2023-01-15

**Authors:** Xiaoyu Chen, Li Cheng

**Affiliations:** 1 Faculty of Education, Beijing Normal University, Beijing 100875, China; 2 Educational and Developmental Research Center of Children’s Creativity, Faculty of Education, Beijing Normal University, Beijing 100875, China

**Keywords:** emotional intelligence, creative self-efficiency, self-esteem, gender differences, gifted children

## Abstract

Creative self-efficacy is a type of individual creative self-belief, which is an important predictor of creative activities and achievements. For gifted children who have high creative potential, the influencing factors of their creative self-efficacy need to be further explored. This study aimed to explore the relationship between gender, emotional intelligence, self-esteem, and creative self-efficacy in gifted children, with special attention on the mediating role of self-esteem and the moderating role of gender in the relationship between emotional intelligence and creative self-efficacy. Participants in this study included 226 gifted students aged 10–13 (135 boys and 91 girls) from experimental classrooms designed for gifted students in North China. The creative self-efficacy scale (CSE), the emotional intelligence scale (EIS), and the self-esteem scale (SES) were tested. The statistical results indicate that: (1) emotional intelligence significantly positively predicted creative self-efficacy in gifted children; (2) self-esteem partially mediated the link between emotional intelligence and creative self-efficacy; and (3) gender acted as a moderator for the mediation model, in which self-esteem played a complete mediating role in gifted boys, while the mediating role of self-esteem in gifted girls was not significant. The results of this study reveal the influential mechanism of creative self-efficacy in gifted children of different genders and may provide further implications for promoting the creative potential of gifted children.

## 1. Introduction

With the rapid development of the third industrial revolution and knowledge economy in the 21st century, creativity has become an indispensable ability of high-quality human resources (Jauk et al. 2015). One of the most influential international student assessment programs, the Program for International Student Assessment Program (PISA) organized by the OECD, planned to add a new domain, which is a test of creative thinking in 2022, to call on the importance of the cultivation of students’ innovative literacy (OECD 2019). As an important predictor of creativity, creative self-efficacy is a type of creative self-belief, which is an individual’s confidence and belief in one’s own creative ability and is a key factor affecting an individual’s creative output (Karwowski and Lebuda 2013; Mathisen and Bronnick 2009; Stolz et al. 2022; Du et al. 2020; Zhang et al. 2021a). A recent meta-analysis showed that the overall mean relation between creativity and creative self-efficacy was of medium size (r = 0.39), which indicated that the creative behavior relied in some part on individuals’ self-belief of their creative potential (Haase et al. 2018).

Gifted children are children with high IQs, and are superior to normal children in cognitive ability. They usually have high creative potential (Karwowski et al. 2016; Angela and Caterina 2020), but some of them do not show a high creative achievement level (Jauk et al. 2013). This might be because some of them do not have enough creative self-efficacy. Especially in China, most of the gifted children studied in the experimental classroom only for the gifted, and there was greater peer pressure than in regular classrooms, which might lead to a decline in the self-concept of some gifted children, and in turn lead to the lack of self-confidence and a low sense of creative self-efficacy (Duan et al. 2022; Fang et al. 2018). In addition, the gifted education in China focused on students’ academic performance, which might lead to a lack of experience in solving creative problems, resulting in a lower sense of creative self-efficacy (Zhang 2017). However, since the macro education environment is difficult to change in a short period of time, it is necessary to analyze the internal factors affecting the creative self-efficacy of Chinese gifted children from the perspective of individuals, to further provide inspiration for improving their creative self-efficacy.

According to the creative self-belief model constructed by Karwowski et al. (2019), creative self-efficacy is affected by many factors. As a domain-specific self-belief, creative self-efficacy arises from the general self-concept of individuals. Self-esteem is “the evaluative component of the self-concept”, and is considered as the final conclusion of self-evaluation (Chiu 1988), which is closely related to the domain-specific self-concept (Karwowski 2012). Furthermore, the self-concept seems more open to contextual changes and is described as a surface characteristic (Karwowski and Lebuda 2016). As a type of surface characteristic, self-concept is more changeable and influenced by both social and psychological environments during development, which is also suggested by socio-cognitive theories (Bandura 1986). Emotional intelligence is an important psychological construct for the individual’s development, which affects the perception of the emotional environment (Ogurlu 2021). As a unique personal characteristic, emotional intelligence overlaps with an individual’s personality, which seems like a hard-core trait, and would cause the change of the surface self-concept (Karwowski et al. 2013). Hence, emotional intelligence is a key factor in self-esteem and creative self-efficacy. Moreover, gender plays a vital role in the development of creative self-belief, because of the different personality traits in different genders, such as the so-called “male-hubris-female-humility bias” (Karwowski et al. 2015). Hence, gender might influence the relationship between emotional intelligence, self-esteem, and creative self-efficacy.

Therefore, this study aimed to focus on the influencing factors of creative self-efficacy in Chinese gifted children and to examine the relationship between emotional intelligence and creative self-efficacy, including the mediating effect of self-esteem and the moderating effect of gender, to provide inspiration for improving the creative self-efficacy of gifted children and promoting their creativity.

### 1.1. Emotional Intelligence and Creative Self-Efficacy in Gifted Children

As an individuals’ self-evaluation of their ability to complete creative tasks, creative self-efficacy is the result of the combined action of the individual and environmental factors, as well as cognitive and noncognitive factors (Lei and Lei 2022; Tierney and Farmer 2011; Puente-Díaz and Cavazos-Arroyo 2017). From the perspective of the individual, emotional intelligence is an outcome of the combinatory relationship of the cognitive and emotional domains, which is closely related to an individual’s thoughts, feelings, and actions (Ciarrochi et al. 2000; Qualter et al. 2012). Salovey and Mayer (1990) first described emotional intelligence as a type of cognitive ability, and used performance-type tests to evaluate these specific skills, including perceiving emotions, assimilating emotions, understanding emotions, and managing emotions (Mayer et al. 1999). However, some scholars suggested that emotional intelligence is different from traditional intelligence, and viewed it as a mixture of cognitive and non-cognitive attributes based on self-perception (Bar-On 2006; Petrides 2009). This type of framework was named trait emotional intelligence, which emphasizes the correlation between emotional intelligence and personality traits. The trait model depends on self-report inventories, and is investigated as a predictor of well-being and adaption actions (Greenberg 2002; Petrides et al. 2007). The trait emotional intelligence can help individuals tap the emotional information of others and express their own emotions in the process of interpersonal communication, which is important in building a harmonious social atmosphere for themselves (Metaj-Macula 2017) and this will influence the generation of their self-concept, such as creative self-efficacy.

Recent studies have indicated a significant prediction of emotional intelligence on creative self-efficacy (Sánchez-Ruiz et al. 2011; Ferdowsi and Razmi 2022). Xu et al. (2019) used a meta-analysis that summarized 75 studies with a total sample of 18,130 to assess the relationship between emotional intelligence and creativity, and the results showed that there was a statistically significant moderate correlation (r = 0.32, *p* < .01) between these two constructs, and the link was stronger when emotional intelligence and creativity were measured using subjective reports, such as trait emotional intelligence and creative personality. In addition, some studies have found that emotional intelligence significantly predicted general self-efficacy (Chan 2004; Yang et al. 2022), and general self-efficacy is close to creative self-efficacy. Meantime, some studies showed that openness, extraversion, and conscientiousness of the big five personality traits had a significant association with creative self-efficacy (Fino and Sun 2022; Karwowski and Lebuda 2016), and that personality is highly correlated with emotional intelligence (Van der Linden et al. 2012). All of these results suggest that emotional intelligence is positively related to creative self-efficacy.

In the field of gifted education, researchers are increasingly recognizing the importance of social and emotional well-being for gifted children (Mammadov et al. 2018; Lubinski and Benbow 2000). Some studies revealed that gifted children faced more emotional challenges, and had more intense feeling than their peers due to the asynchronous development (Winkler and Voight 2016). With the characteristics of intense emotionality, intense empathy, and strong affective expression, emotional intelligence directly affects whether the gifted individual can effectively cope with emotional challenges and achieve self-development (Bar-On 2007). Therefore, emotional intelligence may have a more important impact on the creative self-efficacy of gifted children. However, the present studies on the relationship between emotional intelligence and creative self-efficacy still lack attention to gifted children. Previous studies only pointed out that the emotional intelligence and creativity of gifted children are better than those of nongifted children (Karwowski et al. 2017; Abdulla Alabbasi et al. 2020, 2021), which made it necessary to further verify the relationship between these two variables in the gifted group. Based on previous studies, we developed the following hypothesis:

**Hypothesis** **1.** *Emotional intelligence will be positively related to creative self-efficacy in gifted children.*

### 1.2. Emotional Intelligence, Creative Self-Efficacy, and Self-Esteem in Gifted Children

Self-esteem refers to a basic need in the process of individual growth. It is the individual’s perception of the difference between the actual self-positioning and the ideal self-positioning, which is an evaluative and emotional experience of one’s self (Seeman 1976).

The two-factor theory of self-esteem states that self-esteem is composed of a sense of competence and a sense of worth (Mruk 1995). As a process of social construction, the sense of worth of self-esteem is formed by the individual’s continuous internalization of external evaluations in contact with important others, and thus social factors have a significant impact on the development of individual self-esteem (Pan 2015; San Martín and Barra 2013). Previous studies have shown that support and companionship from parents, peers, and teachers are beneficial to the development of self-esteem among junior high school students, while peer conflict and selfish behavior hinder the development of self-esteem (Doi et al. 2020; Zuffianò et al. 2016). These results indicate that an individual’s self-esteem is affected by the individual’s willingness to communicate with others, as well as the trait to understand and perceive others. This reveals that there is a certain internal connection between emotional intelligence and individual self-esteem (Villanueva et al. 2022; Casino-García et al. 2021). Cheung et al. (2015) conducted a quantitative study by using the Emotional Intelligence Scale and Self-esteem Scale to test 405 students in Hong Kong, China. The results showed that emotional intelligence significantly predicted self-esteem and that emotional intelligence appeared to be a strong determinant of self-esteem. Therefore, we might conclude that emotional intelligence and self-esteem have a positive correlation in gifted children.

Moreover, based on the investment theory of creativity constructed by Sternberg and Lubart (1991), thinking style and personality traits are important individual factors that affect creativity. Self-esteem, as a noncognitive factor that cannot be ignored, has an important impact on creative performance (Sternberg and Lubart 1991; Zhang et al. 2018). Although fewer studies have explored the relationship between self-esteem and creative self-efficacy directly, many empirical studies have shown the predictive effect of self-esteem on creativity, as well as on self-efficacy. A meta-analysis summarized 24 studies with 3956 participants and pointed out that there was a positive correlation between self-esteem and creativity, and especially a significant positive correlation between self-esteem and creative personality (Deng and Zhang 2011). In addition, there have also been many empirical studies supporting that self-esteem can predict an individual’s self-efficacy (Luo et al. 2022; Yang et al. 2019). Marcionetti and Rossier (2021) conducted a longitudinal study to examine the relationship between self-esteem and self-efficacy by using cross-lagged analysis. The results showed that students’ self-esteem significantly predicted their future general self-efficacy. There is a high correlation between creative self-efficacy and creativity, as well as creative self-efficacy and general self-efficacy. The creative self-efficacy derives from general self-efficacy and further predicts individual creativity (Karwowski et al. 2019; Beghetto 2006). According to this, the relationship between self-esteem and creative self-efficacy can be inferred, that is, self-esteem will positively predict an individual’s creative self-efficacy.

Self-esteem is of great significance to the healthy development of gifted children and affects how individuals view their own abilities, generate behavioral motivation, and regulate their emotions (Sowislo and Orth 2013; Li et al. 2021; Papadopoulos 2021). Compared with nongifted children, gifted children had a higher level of self-concept (Litster and Roberts 2011) and had higher achievement expectations for themselves. Based on the importance of self-esteem to gifted children and the differences between gifted children and nongifted children, it is necessary to further explore the influence mechanism of self-esteem in the relationship between emotional intelligence and creative efficacy of gifted children. According to previous studies, we concluded that self-esteem has a predictive effect on creative self-efficacy in gifted children. Therefore, we developed the following hypothesis:

**Hypothesis** **2.** *Self-esteem will play a mediating role in the relationship between emotional intelligence and creative self-efficacy in gifted children.*

### 1.3. Gender Differences among Emotional Intelligence, Creative Self-Efficacy, and Self-Esteem in Gifted Children

Gender differences have always been a primary topic of concern for researchers focused on gifted children. Previous studies have found that gifted girls have significantly higher scores in emotional intelligence than gifted boys (Schwean et al. 2006; Ogurlu 2021), while the self-efficacy and self-esteem of gifted boys are significantly higher than those of gifted girls (Sarouphim 2011; Su et al. 2010; Junge and Dretzke 1995). However, some studies have reached different conclusions, arguing that there are no significant gender differences in these three variables among gifted children (Singh and Kaur 2012; Topçu and Leana-Taşcılar 2018; Chan 2005). Based on this, we will further explore the gender differences of gifted children’s creative self-efficacy, emotional intelligence, and self-esteem.

These uncertain gender differences in the three variables might further affect the specific performance of the pathways of emotional intelligence, self-esteem, and creative self-efficacy in different genders and make gender moderate the mediation model of self-esteem. In addition, previous studies have shown that different gender expectation would affect individuals’ behavior. Society prefers to accept gifted boys’ individualism and independence while expecting girls to be rather cooperative and show empathy in interpersonal relationships (Bianco et al. 2011). Due to the influence of gender expectations, gifted girls are more likely to be dependent on the external environment and more sensitive to emotional changes, and their self-concept is more easily affected by external evaluations, while boys tend to obtain resources internally and tend to solve problems independently (Milfont and Sibley 2016). This might also lead to differences in the mediating effect of self-esteem between emotional intelligence and creative self-efficacy between gifted boys and girls.

According to the above, the moderation effect of gender in the relationship between emotional intelligence and creative efficacy, emotional intelligence and self-esteem, and self-esteem and creative self-efficacy in gifted children cannot be accurately inferred according to previous studies, so this study used an exploratory approach to analyze the moderating role of gender in these three pathways at the same time, so as to analyze the gender difference in the mediating effect of self-esteem. Therefore, we developed the following hypothesis:

**Hypothesis** **3.** *Gender will play a moderating role in the mediating model of self-esteem in the relationship between emotional intelligence and creative self-efficacy in gifted children.*

Creative self-efficacy is of great significance to the development of the creative potential of gifted children, so it is necessary to focus on the influencing factors of creative self-efficacy on it. In conclusion, emotional intelligence, self-esteem, and gender are key influencing factors of creative self-efficacy of gifted children.

This study focused on the construction of creative self-efficacy among gifted children in the Chinese context, so we also considered the specificities of creative self-efficacy in the Chinese context (Zhang et al. 2021b), and found that the effects of emotional intelligence, self-esteem, and gender on the creative self-efficacy of gifted children in the Chinese context may be more important. Studies have shown that the Chinese unique cultural environment, made up of elements such as the Confucian tradition, the one-child policy, and son preference, affects individual creativity (Guo et al. 2018). Creative self-efficacy, as an individual’s perception of their own creative ability, is also influenced by the Chinese cultural environment. First, China pursues the concept of collectivism and emphasizes the integration of the collective, which makes the individual’s self-efficacy more derived from the perception of external support (Lin et al. 2017). As an important trait of individuals dealing with emotional states, emotional intelligence plays an important role in the interaction between individuals and the outside world, so it is necessary to emphasize the unique impact of emotional intelligence on creative self-efficacy in the Chinese context. Second, since ancient times, China has focused on self-reflection, emphasizing the personal virtue of humbleness, requiring individuals to have a clear understanding of themselves and not to be arrogant and boastful. As a type of self-evaluation, self-esteem is regarded by Chinese as one of the most important characteristics, which profoundly affects the thinking and behavior of individuals (Li et al. 2015), so considering self-esteem is one of the important factors affecting Chinese creative self-efficacy. Finally, the traditional gender stereotypes and gender expectations of Chinese society profoundly affect the development of children (Zhao et al. 2022; Ju et al. 2015). Chinese parents generally believe that “boys are smart, girls are diligent”, “boys should be independent, girls should rely on others”, which will subtly affect individual development, resulting in different development trajectories and influence mechanisms for children’s creative efficacy of different genders. Therefore, the moderating role of gender also needs to be considered in the Chinese context.

**Figure 1:**
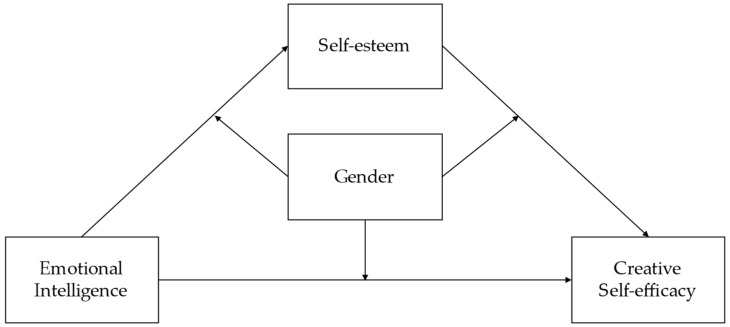
The hypothesized model.

Combined with the analysis of the developmental environment of gifted children in the Chinese context, this study aimed to explore the relationship between emotional intelligence and creative self-efficacy in Chinese gifted children. Furthermore, we hypothesized that this relationship would be mediated by self-esteem and that gender would moderate this mediation model (Figure 1). To test these hypotheses, we first conducted a bivariate analysis of emotional intelligence, self-esteem, creative self-efficacy, and gender. Then, we investigated the mediating effect of self-esteem and the moderating effect of gender by using structure equation modeling.

## 2. Materials and Methods

### 2.1. Participants

The participants for this study were recruited from experimental classrooms for gifted students in North China. Children in these classrooms were identified as gifted through a three-step strategy, including preliminary testing, retesting, and dynamic evaluation. A comprehensive evaluation of students’ cognitive ability, academic level, learning ability, development potential and other aspects was used to identify gifted children based on the Three Ring Conception of Giftedness proposed by Renzulli (1978) and the WICS model proposed by Sternberg (2003).

A total of 231 gifted children aged 10–13 years were enrolled in this study and completed the questionnaire. After eliminating the unqualified forms (those with obvious false answers and those with contradictory answers to forward and backwards scoring items), 226 valid questionnaires were finally obtained, which were 97.84% effective. The final participants were comprised of 135 boys (59.73%) and 91 girls (40.27%). The average age of all participants was 11.44 years (SD = 0.71), of which boys’ average age was 11.40 years (SD = 0.73) and girls’ average age was 11.49 years (SD = 0.66).

### 2.2. Materials

#### 2.2.1. Creative Self-Efficacy Scale (CSE)

The Creative Self-Efficacy Scale (CSE) compiled by Beghetto (2006) was used to measure the creative self-efficacy of gifted children. The scale consists of three items and is a one-dimensional scale (e.g., “I have a lot of good ideas.”). We first translated the items into Chinese and then invited a postgraduate student majoring in special education to reverse-translate the Chinese items back to English with no prior knowledge of the original statements. The back-translation items were compared with the original scale one by one, and it was found that the back-translation items were basically consistent with the original items, indicating that the Chinese translation items were basically accurate. Participants were asked to rate these items on a 5-point scale ranging from 1 (strongly disagree) to 5 (strongly agree). Then, the average score of all items was calculated, with higher scores representing higher levels of creative self-efficacy. In this study, the Cronbach’s alpha coefficient of the Chinese version scale was 0.87, with high internal consistency.

#### 2.2.2. Emotional Intelligence Scale (EIS)

The Emotional Intelligence Scale (EIS) was first compiled by Schutte et al. (1998), and the Chinese version was compiled by Wang and He (2002). We used the Chinese version to examine the emotional intelligence of the participants. It is a self-report scale with a total of 33 items divided into 4 dimensions, including emotion perception (10 items, e.g., “I am aware of my emotions as I experience them”), managing self-relevant emotions (9 items, e.g., “I seek out activities that make me happy”), managing others’ emotions (8 items, e.g., “I arrange events others enjoy”), and emotion utilization (6 items, e.g., “When I feel a change in emotions, I tend to come up with new ideas”). Participants were asked to rate these items on a 5-point scale ranging from 1 (strongly disagree) to 5 (strongly agree). Three items were scored backwards (e.g., “I find it hard to understand the nonverbal messages of other people”), and the average score of each dimension and the total scale were then calculated, with higher scores representing higher levels of emotional intelligence. In this study, the Cronbach’s alpha coefficient of the total scale was 0.93, and the Cronbach’s alpha coefficients of the four dimensions of emotion perception, managing self-relevant emotions, managing others’ emotions, and emotion utilization were 0.86, 0.86, 0.67, and 0.83, respectively.

#### 2.2.3. Self-Esteem Scale (SES)

The Self-esteem Scale (SES) was first developed by Rosenberg et al. (1978), and we used its Chinese version (Peng et al. 2021) to measure the self-esteem of the participants. It is a self-reported single-dimensional scale, with a total of 10 items, of which 5 items are forward-scoring items and five are backwards-scoring. Participants were asked to rate these items on a 5-point scale ranging from 1 (strongly disagree) to 5 (strongly agree), and the average score of all items was calculated, with higher scores representing higher levels of self-esteem. In this study, the Cronbach’s alpha coefficient of the Chinese version scale was 0.84, with high internal consistency.

### 2.3. Procedures

This study was approved by the Research Ethics Committee of the Faculty of Education, Beijing Normal University (protocol code: BNU202106100016). The measures were administered by postgraduate students majoring in special education, and all of them were well-trained by the researchers before the data collection. The test administration steps were clarified for experimenters and each was provided with detailed instructions. We adopted an offline paper-and-pencil group test, and all participants were asked to complete the questionnaire independently. At the beginning of the questionnaire, a written informed consent form was provided to all participants and their guardians. The purpose of the study was stated in the form, and participants were informed that the study was confidential and that they may opt out at any time. After signing the informed consent form, the participants were invited to complete all questionnaires, which took approximately 25 min.

### 2.4. Data Analysis

This study aimed to explore the role of self-esteem and gender in the relationship between emotional intelligence and creative self-efficacy. Accordingly, Pearson’s correlation analysis and the independent sample *t* test were first conducted in SPSS 26.0 to analyze the correlations between emotional intelligence, self-esteem, and creative self-efficacy and the gender differences in these three characteristics. Then, structure equation modeling was performed in Mplus 8.3 to test the mediating effect of self-esteem on the association between emotional intelligence and creative self-efficacy. Finally, a multigroup model comparison was performed in Mplus 8.3 to test the moderating effect of gender in the mediation model of self-esteem.

When performing the structure equation modeling, the following criteria were used to determine whether the data fit the hypothesized model well: (1) χ^2^/df < 3.00; (2) CFI > 0.90; (3) TLI > 0.90; (4) RMSEA < 0.08; (5) SRMR < 0.08 (Hu and Bentler 1999; Li et al. 2022).

Additionally, the bootstrapping method was applied to test the significance of the mediation effects to obtain robust standard errors for parameter estimation (Chen et al. 2022a). This method produced 95% bias-corrected confidence intervals (CIs) for these effects from 2000 resamples of the data. CIs that do not contain zero indicate significant effects.

## 3. Results

### 3.1. Bivarite Analysis

The means, standard deviations, and correlations among variables are presented in Table 1. The total score of emotional intelligence and its four dimensions scores of gifted children were significantly positively correlated with creative self-efficacy and self-esteem at the 0.001 significance level, and self-esteem was also positively correlated with creative self-efficacy at the 0.001 significance level. In addition, age had a significant negative correlation with creative self-efficacy (r = −0.17, *p* < .01), indicating that age should be used as a control variable in further mediation model testing.

Furthermore, an independent sample *t* test showed that the creative self-efficacy of gifted boys (M = 3.98, SD = 0.93) was significantly higher than that of girls (M = 3.62, SD = 0.81), t_(224/2)_ = 3.07, *p* < .01, Cohen’s d = 0.41. There were no significant gender differences in the emotional intelligence and self-esteem scores of gifted children.

### 3.2. The Mediating Effect of Self-Esteem on the Relationship between Emotional Intelligence and Creative Self-Efficacy

The four dimensions of emotional intelligence had significant positive correlations with creative self-efficacy and self-esteem. According to the requirement of mediating effect testing suggested by Baron and Kenny (1986), which was that the correlation among variables was the premise to test the mediating effect, we took the observed variable creative self-efficacy as the dependent variable, the latent variable emotional intelligence as the independent variable, the observed variable self-esteem as the mediating variable, and the observed variable age as the control variable. Then, the mediation model was constructed using Mplus8.1.

Based on the mediation effect test strategy proposed by Wen and Ye (2014), we first conducted a non-mediating model to test the total effect of emotional intelligence on creative self-efficacy. The results of the model fit indices show that the data fit the hypothetical model (χ^2^/df = 2.55, CFI = 0.98, TLI = 0.97, RMSEA = 0.08, SRMR = 0.03), and all of the normalized path coefficients in the model reached the 0.001 significance level. After controlling for age, emotional intelligence significantly positively predicted gifted children’s creative self-efficacy (β = 0.52, *p* < .001), which showed that the total effect was significant.

**Figure 2:**
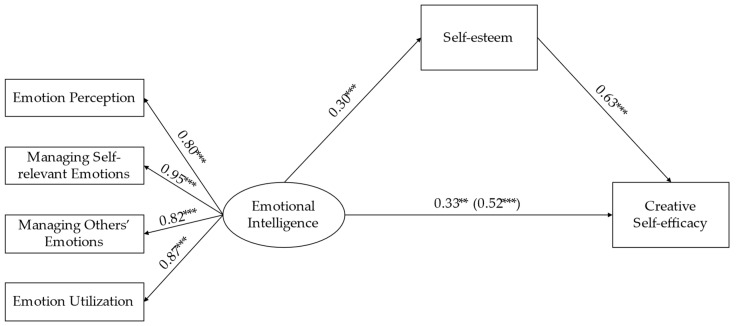
The mediating effect of self-esteem on the relationship between emotional intelligence and creative self-efficacy. ** *p* < .01, *** *p* < .001; The numbers are standardized regression coefficients. The residuals variances and controlled variable (age) in the model are not displayed.

Then, we constructed the mediating model to test the mediating effect of self-esteem on the relationship between emotional intelligence and creative self-efficacy. The results of the model fit indices show that the data fit the hypothetical model (χ^2^/df = 2.29, CFI = 0.98, TLI = 0.97, RMSEA = 0.08, SRMR = 0.03), and all of the normalized path coefficients in the model reached the 0.01 significance level (see Figure 2). After controlling for age, emotional intelligence still significantly positively predicted gifted children’s creative self-efficacy (β = 0.33, *p* < .01), which showed that the direct effect of the mediating model was significant. Furthermore, emotional intelligence significantly positively predicted gifted children’s self-esteem (β = 0.30, *p* < .001), and self-esteem significantly positively predicted their creative self-efficacy (β = 0.63, *p* < .001). The bootstrapping method was used to test the mediating effect of self-esteem, and the result shows that the mediating effect of self-esteem between emotional intelligence and creative self-efficacy was significant (95% CI [0.10, 0.31], excluding 0). The mediating effect value was 0.19, accounting for 36.54% of the total effect, indicating that self-esteem played a partial mediating role between emotional intelligence and creative self-efficacy.

### 3.3. Moderating Effect of Gender in the Mediation Model of Self-Esteem

To explore the moderating role of gender in the mediating model of emotional intelligence, self-esteem, and creative self-efficacy in gifted children, we used the multigroup model comparison in Mplus 8.1 to analyze whether there was a gender difference in the mediating effect of self-esteem between emotional intelligence and creative self-efficacy of gifted children.

Based on the above mediation model, we first constructed the boy model (Model 1) and girl model (Model 2) independently. The results of both models’ fit indices show that the data fit the hypothetical model (see Table 2), which indicated that it was possible to further construct the model comparison. Second, we constructed the unrestricted mediation model of self-esteem in the relationship between emotional intelligence and creative self-efficacy (Model 3), and on this basis, we constructed the restrictive model with equal regression coefficients across groups (Model 4). The results of the model fit indices show that the data fit the hypothetical model in both Models 3 and 4 (see Table 2). Furthermore, according to Δχ^2^ and Δdf, to judge the significance of the model difference, we found that there was a significant difference between the unrestricted model and the restricted model (Δχ^2^ (1) = 5.51, *p* < .05), which showed that the restrictive model was better, indicating that there was a significant gender difference in the mediating effect of self-esteem between emotional intelligence and creative self-efficacy in gifted children. This result suggests that the moderating effect of gender was significant in the mediation model of self-esteem.

**Figure 3:**
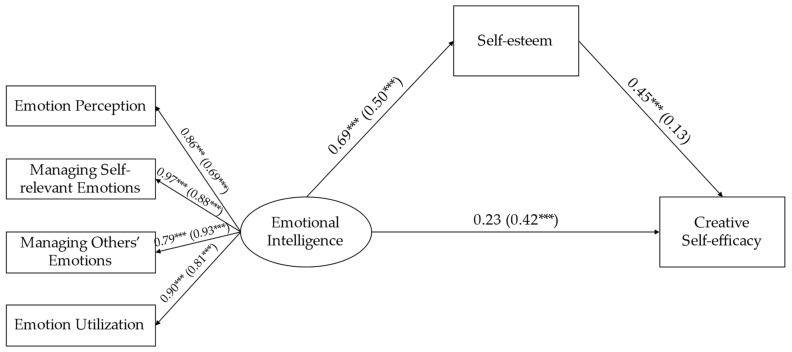
A multigroup model comparison of the mediating effect of self-esteem. *** *p* < .001; The numbers are standardized regression coefficients, which boys’ coefficients are outside brackets, and girls’ coefficients are inside brackets. The residuals variances and controlled variable (age) in the model are not displayed.

Then, we constructed a specific analysis of the path coefficients of the boy model and the girl model (see Figure 3). Emotional intelligence positively predicted self-esteem in both the boy and girl groups of gifted children (boy: β = 0.69, *p* < .001; girl: β = 0.50, *p* < .001). Self-esteem significantly positively predicted creative self-efficacy in the boy group but had no significant predictive effect in the girl group (boy: β = 0.45, *p* < .001; girl: β = 0.13, *p* > .05). Emotional intelligence had no significant predictive effect on creative self-efficacy in the boy group but had a significant positive predictive effect in the girl group (boy: β = 0.23, *p* > .05; girl: β = 0.42, *p* < .001). The bootstrapping method was used to test the mediating effect of the self-esteem of gifted children of different genders, and the results show that the mediating effect of self-esteem between emotional intelligence and creative self-efficacy was 0.31 in gifted boys, which was significant (95% CI [0.18, 0.48], excluding 0), accounting for 57.41% of the total effect. Meanwhile, the mediating effect of self-esteem between emotional intelligence and creative self-efficacy was 0.07 in gifted girls, which was not significant (95% CI [−0.02, 0.20], including 0), accounting for 14.29% of the total effect. The results indicate that self-esteem plays a complete mediating role between emotional intelligence and creative self-efficacy in gifted boys, while the mediating effect did not exist in gifted girls.

## 4. Discussion

In this study, we investigated the relationship between emotional intelligence and creative self-efficacy in Chinese gifted children and further focused on the mediation effect of self-esteem and the moderation effect of gender. The results indicate that emotional intelligence significantly positively predicted creative self-efficacy in gifted children, and this relationship was partially mediated by self-esteem. In addition, the mediating effect of self-esteem had a significant gender difference, which shows that gender played a moderating role in the mediation model.

### 4.1. The Effect of Emotional Intelligence on Creative Self-Efficacy

The results show that emotional intelligence significantly positively predicted creative self-efficacy in gifted children, which supports Hypothesis 1.

As an individual psychological trait, emotional intelligence affects the individual’s self-construction in the interaction with the external environment (Petrides and Furnham 2001; Schutte et al. 2002), which in turn affects the individual’s creative self-efficacy. The result of this study validates social cognitive theories (Bandura 1986) and supports previous findings that individual personality traits are significantly associated with creative self-efficacy (Fino and Sun 2022; Karwowski and Lebuda 2016). Emotional intelligence, as an individual’s trait to interact emotionally with others, profoundly affects an individual’s perception and integration of the external environment (Herland 2022). Previous studies have shown that emotional intelligence has a significant predictive effect on interpersonal relationships. Individuals with high emotional intelligence had higher social skills and were more able to establish high-quality interpersonal relationships (Chen et al. 2017), which was beneficial for them to obtain support and encouragement from their family, peers, and society. This type of supportive environment could make individuals more confident in their creative activities and enhance their creative self-efficacy (Capron Puozzo and Audrin 2021).

This study also enriched the researches on emotional intelligence and creativity. Studies have found the predictive effect of emotional intelligence on creativity (Sánchez-Ruiz et al. 2011; Ferdowsi and Razmi 2022; Xu et al. 2019), and this study further named the importance of emotional intelligence for creative self-efficacy, revealing the close relationship between creative self-efficacy and creativity (Karwowski 2012; Pretz and McCollum 2014). According to the systems model of creativity, creativity is influenced by person factors, field factors, and domain factors (Csikszentmihalyi 1997). Creative self-efficacy, as an individuals’ belief in their ability to solve creative problems, belongs to the person factor and plays an important role in the creative process of individuals.

Based on the exploration of the relationship between emotional intelligence and creative self-efficacy, this study especially focused on the group of gifted children. The predictive effect of emotional intelligence on creative self-efficacy in gifted children suggests that emotional intelligence is of great significance to the healthy development of gifted children (Eklund et al. 2015). In the past few decades, the large amount of studies on the cognitive abilities of gifted children has far surpassed the studies on the emotional domains, which has led to the neglect of the emotional characteristics of gifted children (Ogurlu 2020). In recent years, researchers have pointed out that in addition to cognitive factors, social and psychological factors can also influence the dynamic growth path of gifted children (Zeidner 2017). As a trait that generates, recognizes, perceives, and expresses emotions, emotional intelligence affects whether gifted children can accurately understand themselves and the environment, and determine whether they can become a person who contributes to society (Pfeiffer 2001). According to the Differentiated Model of Giftedness and Talent constructed by Gagné (2004), whether an individual’s giftedness translates into talent depends on a variety of factors, including the environment and internal catalysts. Emotional intelligence is an important internal factor that influences the interaction of gifted children with the external environment. Higher emotional intelligence can help gifted children grasp the support and opportunities in the environment, thereby promoting the transformation of talents and achieving individual development. In the process of development, gifted individuals will continue to accumulate the confidence brought by successful experience, thereby improving their sense of confidence. Therefore, emotional intelligence has a significant predictive effect on the creative self-efficacy of supernormal children.

In addition, this study also focused on the important role of emotional intelligence on the creative self-efficacy of gifted children in the Chinese context. Under the influence of Chinese collectivism and Confucianism, individuals need to observe etiquette, treat others kindly, and seek ideological and emotional consistency with the collective (Oyserman et al. 2002). Especially when solving creative problems, Chinese are likely to discuss and negotiate in a group (Niu and Kaufman 2013). In this context, individuals with high emotional intelligence can better interact with others, better understand others’ views, and express their own opinions. This helps them to better participate in collective decision-making and gain confidence in solving creative problems, thus enhancing their creative efficacy.

Thus, we can enhance the creative self-efficacy of Chinese gifted children by developing their emotional intelligence. We can use group counselling, club activities, sand play therapy, and other strategies to improve the ability of gifted children’s emotional perception, emotional reasoning, and emotional expression, helping them create good interpersonal relationships and emotional environments, thereby enhancing their creative self-efficacy.

### 4.2. The Mediating Effect of Self-Esteem

This study further showed that the relationship between emotional intelligence and creative self-efficacy in gifted children was partially mediated by self-esteem, which supports Hypothesis 2. Emotional intelligence, as a hard-core trait, affects the surface general self-concept characteristics, which in turn affects specific-domain self-concepts, such as creative self-efficacy, validating the creative self-belief model constructed by Karwowski et al. (2019).

First, we found that self-esteem was affected by individual emotional intelligence, which was similar to previous studies (Villanueva et al. 2022; Cheung et al. 2015). The self-verification theory of self-esteem states that self-esteem is closely related to the social environment and information processing preferences. Driven by the need for consistency in self-verification, when the evaluative information obtained from the external environment is consistent with self-expectation, the individual’s self-esteem is enhanced (He et al. 2005; Swann 1999). Emotional intelligence, as an important psychological resource for individuals to process emotional information, has a significant impact on individuals’ acquisition and understanding of external evaluation information. Individuals with higher emotional intelligence had more resources to manage and process other people’s and their own emotions and could use their own advantages in emotional perception to seek more positive communication experience and interactive feedback. This could help them obtain more evaluation information consistent with self-expectation from the external environment, thereby improving their self-esteem level (Gao et al. 2021; Feng et al. 2012).

We also found that self-esteem had a significant predictive effect on individual creative self-efficacy, which was also similar to previous findings (Barbot 2020; Chen et al. 2022b; Deng and Zhang 2011). Individuals with high self-esteem viewed their abilities more positively, which helped them better affirm their self-worth and have more confidence in completing difficult creative tasks (Luo et al. 2022; Christy and Mythili 2020). Based on Maslow’s hierarchy of needs theory, esteem need was the basis of self-fulfillment needs, and creative self-efficacy was one of the forms of self-fulfillment needs (Healy 2016). With the improvement of self-esteem level, the individual’s esteem need was constantly being satisfied, and then the need for self-fulfillment was gradually generated. In the process of pursuing self-fulfillment, individuals can constantly stimulate their creative potential, experience success in challenging creative tasks, and then have a higher sense of creative self-efficacy.

The results of this study reveal the important mediator role of self-esteem in the relationship between emotional intelligence and creative self-efficacy in gifted children. First, gifted children were more sensitive than their nongifted peers, and their self-construction mostly depended on external evaluation (Gere et al. 2009). Emotional intelligence helps gifted individuals to understand others’ emotions and express their own emotional experience, and affects the unity of self-evaluation and others’ evaluation of gifted children, which also affects their self-esteem level (Casino-García et al. 2021). In addition, previous studies showed that gifted children have a stronger ability to understand and a more accurate self-concept (Litster and Roberts 2011; Duan et al. 2022). As a core aspect of self-concept, the high self-esteem of gifted children makes them have high performance expectations for themselves (Olton-Weber et al. 2020; Mofield and Peters 2019). Those with high self-esteem believed that they can achieve their goals and then face challenges and difficulties with a more positive attitude, and have higher confidence in their ability to complete creative tasks (Topçu and Leana-Taşcılar 2018). Those with low self-esteem thought that they could not meet their expectations and then developed feelings of depression and loss and gradually lost confidence and courage to challenge creative tasks. Thus, self-esteem plays a mediating role in the relationship of emotional intelligence and creative self-efficacy of gifted children.

In addition, the results of this study also support the key role of self-esteem in the construction of individual creative self-efficacy in the Chinese context. In traditional Chinese culture, individuals are required to have a clear assessment of themselves and to continuously improve themselves through self-reflection (Huang and Li 2006). As a product of individual self-evaluation, self-esteem comes from the perception of external environmental information, is affected by the psychological characteristics, and then has an important impact on the self-development and self-fulfillment of Chinese people. Based on this, self-esteem plays an important mediating role in the relationship between emotional intelligence and creative self-efficacy in the Chinese context.

Therefore, we need to continuously improve the self-cognition of Chinese gifted children, help them experience a greater sense of achievement and value, promote their self-esteem, and finally achieve the purpose of improving their creative self-efficacy.

### 4.3. The Moderating Effect of Gender

Furthermore, this study found that there were differences in the mechanism of emotional intelligence on creative self-efficacy in gifted children of different genders, illustrating that gender played a moderating role in the mediation model, supporting Hypothesis 3.

The independent sample *t* test indicated that the creative self-efficacy of gifted boys was significantly higher than that of girls, which is consistent with the findings of previous studies (Pajares 1996; Junge and Dretzke 1995). Gifted boys had higher self-confidence and a stronger sense of identity in their ability to complete creative tasks and solve creative problems. This might be related to the fact that gifted boys can often experience a sense of success in completing difficult learning tasks. Especially in the Chinese context, gifted students studied in the experimental classrooms, where more attention was given to the study of mathematics and physics, emphasizing the deepening of learning difficulty in these subjects. Gifted children were asked to finish challenging learning tasks, conduct divergent thinking, and put forward their own creative ideas (Liu 2022). Gifted boys who had stronger mathematical thinking ability than girls were often more able to solve difficult learning problems, had a more positive learning attitude in these subjects, and thus had better performance (Deringöl 2018). During this process, Chinese gifted boys continued to receive positive feedback and experienced their own abilities and a sense of control over tasks. This accumulation of confidence and belief might help them have higher creative self-efficacy than girls. Therefore, we should pay more attention to the creative self-efficacy of Chinese gifted girls, provide opportunities for them to display their advantages, and continuously make them accumulate successful experience by improving the curriculum system and expanding teaching methods to improve their self-confidence in completing creative tasks.

Additionally, the results of the multigroup model comparison showed that self-esteem played a complete mediating role between emotional intelligence and creative self-efficacy in gifted boys, and the mediating effect of self-esteem was not significant in gifted girls. This might be caused by traditional cultural concepts and social expectations (Li et al. 2022; Liu 2021). Especially in the Chinese context, there is a proverb called “Boys should be self-reliant”, that is, boys are expected to become more independent. At the same time, under the son preference, boys are usually put to higher demands (Chu et al. 2007). Under this gender expectation, boys are more independent and tend to solve problems by themselves compared with girls (Nie et al. 2017; Milfont and Sibley 2016). In particular, gifted boys had higher requirements for themselves, emphasized the mobilization of internal psychological resources, and rarely sought external support (French et al. 2011). This made the self-awareness of gifted boys play a more important role in their psychological behaviors. Gifted boys with high self-esteem had good self-regulation ability and had strong motivation and willingness to approach high-difficulty creative tasks and were much more confident in their own creative ability. Gifted boys with low self-esteem lack effective psychological resource support, which leads to lower creative self-efficacy. Compared with boys, the society’s expectation of girls in the Chinese context is to be considerate and caring for others, believing that girls are better able to handle interpersonal relationships and can profit from interpersonal interactions. Hence, girls tend to pay more attention to interpersonal communication and are more sensitive to emotional changes in others and themselves (Ciarrochi et al. 2005; Zhang and Liu 2021). Similar to nongifted girls, gifted girls were also highly dependent on social relationships and had a strong sensitivity to changes in the external environment (Kao 2011). If gifted girls could obtain sufficient emotional support and create a good emotional environment for themselves, their perception of ability and related behavioral performance would be directly enhanced. Therefore, emotional intelligence, as the ability to perceive and process emotional information, had a significant and direct predictive effect on the creative self-efficacy of gifted girls, and the mediating effect of self-esteem was not significant.

Therefore, based on their different influence mechanisms of creative self-efficacy, we should provide differentiated support to Chinese gifted children of different genders. For gifted boys, we should support them to continuously improve their positive self-concept and improve their self-esteem. Meanwhile, we should help them experience the fun of cooperation and interpersonal communication and encourage them to use external resources to support and solve problems to increase their confidence. For Chinese gifted girls, we should develop their emotional intelligence level so that they can better benefit from emotional communication and interpersonal dependence. Additionally, we should encourage their interest in independent exploration and help them obtain support from their own psychological resources to improve their self-esteem and self-confidence. By providing a targeted and differentiated teaching strategy for gifted boys and girls, the goal of improving creative self-efficacy for both boys and girls can be achieved through multiple channels. Finally, we also need to fight gender stereotypes. Especially in China, which is developing rapidly, we should further promote the development of gender equality, view the development of gifted children from a more inclusive perspective, encourage gifted children to choose their own development path, and then help to construct their creative self-efficacy.

## 5. Conclusions

The present study was designed to explore the relationship between emotional intelligence and creative self-efficacy in Chinese gifted children, especially focusing on the mediating effect of self-esteem and the moderating effect of gender. We found that emotional intelligence significantly positively predicted emotional intelligence and creative self-efficacy, and self-esteem played a partial mediating role in this link. Meanwhile, a gender difference was found in the mediating effect of self-esteem, of which self-esteem played a complete mediating role in gifted boys, while the mediating role of self-esteem in gifted girls was not significant.

Gifted children possess unique advantages in both cognitive and affective elements, which give them high creative potential. The results of this study are of great significance for improving the creative self-efficacy of gifted children, stimulating their creative potential and promoting their creative achievements.

Additionally, there are several limitations in this study. The first and main limitation lies in the cross-sectional design and by solely relying on self-report. In future studies, we should conduct longitudinal studies to further identify causal associations between emotional intelligence, self-esteem, and creative self-efficacy. In addition, we can use various methods to collect data from different perspectives, combining subjective reports with objective tests, to obtain more comprehensive and reliable results. Another potential limitation is the sample characteristic. This study selected a unique sample group with a particular cultural context, so the cross-culture design needs to be conducted in future studies, as well as selecting nongifted children as participants.

## Figures and Tables

**Table 1:** Means (M), standard deviations (SD), and correlations among variables.

Viriables	M	SD	1	2	3	4	5	6
1 Creative self-efficacy	3.83	0.90						
2 Emotion perception	3.69	0.73	0.42 ***					
3 Managing self-relevant emotions	3.88	0.71	0.47 ***	0.76 ***				
4 Managing others’ emotions	3.85	0.88	0.39 ***	0.70 ***	0.77 ***			
5 Emotion utilization	3.86	0.70	0.52 ***	0.66 ***	0.83 ***	0.73 ***		
6 Emotional Intelligence	3.82	0.67	0.50 ***	0.90 ***	0.92 ***	0.87 ***	0.88 ***	
7 Self-esteem	3.03	0.55	0.51 ***	0.54 ***	0.61 ***	0.46 ***	0.55 ***	0.60 ***
8 Age	11.44	0.71	−0.17 **	0.00	0.00	0.07	−0.03	−0.02

Note: ** *p* < .01, *** *p* < .001.

**Table 2:** Model fit indices in multigroup model comparison.

Model	χ^2^/df	CFI	TLI	RMSEA	SRMR
Model 1	1.80	0.98	0.97	0.08	0.04
Model 2	1.30	0.99	0.98	0.06	0.07
Model 3	1.43	0.98	0.98	0.06	0.06
Model 4	1.55	0.98	0.97	0.07	0.06

## Data Availability

The data are currently not publicly available due to participant privacy, but they are available from the corresponding author upon reasonable request.
